# Supplementary material for: The development of numerical mapping in preschool children
Source: PLoS One. 2025 Jun 27;20(6):e0325209. doi: 10.1371/journal.pone.0325209 (PMC12204520; doi:10.1371/journal.pone.0325209)
Supplement: Attachment — Submitted filename: Response to Reviewers.docx [file pone.0325209.s002.docx]

Dear editor and dear reviewers

Re: Manuscript ID: PONE-D-25-02198 and Title: “The development of numerical mapping in preschool children”

Thank you for your letter and for the reviewers’ insightful comments regarding our manuscript. We are deeply grateful for your careful review and thoughtful suggestions, which have substantially improved the quality of our paper.

We have uploaded the revised manuscript. All revisions in the text are marked in red highlight for easy reference. Our detailed responses to the reviewers’ comments are also provided, with changes clearly highlighted. To facilitate access to our data, we have uploaded the stimuli, raw data, and related experimental materials to the Open Science Framework at <https://osf.io/rs2up/>. Additionally, I have updated my information in Editorial Manager and validated my ORCID iD.

This work was supported by the National Natural Science Foundation of China (grant number 32360203) and the Major Project of Key Research Bases of Humanities and Social Sciences of the Ministry of Education (grant number 22JJD10009). The funders had no role in study design, data collection and analysis, decision to publish, or preparation of the manuscript. As requested, we have included the full name of the ethics committee in the Methods section and have attached the official stamped ethics approval document.

We would love to thank you for allowing us to resubmit a revised copy of the manuscript and we highly appreciate your time and consideration.

Sincerely.

Yun Pan.

Point-by-point responses to reviewers' comments are as follows:

**Reviewer 1**

**1. Data Availability
As per PLOS Data Policy, all data underlying the findings should be made available without restriction, unless there are specific reasons for withholding it. The manuscript states that readers must "reach out to the corresponding author with a reasonable request" to obtain the data. Please provide a clear justification for why the data cannot be shared publicly and why restrictions on sharing exist.**

**Response：**Thank you for your precious comments and advice. To facilitate readers to obtain data, we have uploaded the stimuli, raw data and related experimental materials into the Open Science Framework at https://osf.io/rs2up/.

**2. Age Grouping Justification
The manuscript presents age groupings of 2-, 3-, and 4-year-olds without providing a rationale for these specific categories. It would be beneficial to explain why these particular age groupings were chosen and how they align with relevant developmental stages to support the robustness of the study design.**

**Response：**Thank you for your precious comments and advice. To ensure our sample is comparable to that of Benoit et al. (2013), we divided the children into three age groups based on the sample characteristics reported in their study. Additionally, following the presentation of average age in Benoit et al. (2013), we have revised the way we report average age accordingly, as indicated in red in the manuscript (Page 10, Lines 209-213).

**Revised Text in Manuscript:** “To make our sample as comparable to that of Benoit et al. (2013) we further divided the children into three age groups : 40 two-year-olds (20 male; range = 24–35 months; M = 2 years 7 months), 45 three-year-olds (23 male; range = 36–47 months; M = 3 years 7 months), and 37 four-year-olds (20 male; range = 48–59 months; M = 4 years 4 months).”

**3. Sampling Strategy**
**While the geographic locations of participant recruitment are mentioned, the manuscript lacks a description of the sampling strategy. It remains unclear whether this was a convenience sample or if any selection criteria were applied. Please provide more detail on the recruitment process, including whether participants were randomly selected or chosen based on particular inclusion/exclusion criteria. This additional information will help assess potential biases in the sampling method and improve the transparency of the study design.**

**Response：**Thank you for your precious comments and advice. In response, we have revised the manuscript to provide a clearer description of the participant recruitment process. Specifically, we now state that a stratified random sampling method was used within each preschool to ensure both representativeness and gender balance among participants. This revision appears in the *Methods* section (Pages 10-11, Lines 215-216).

**Revised Text in Manuscript:** “A stratified random sampling method was employed within each preschool, ensuring equal representation of boys and girls across the selected age groups.”

**4. Control Conditions
In Experiment 1, the manuscript does not specify what control conditions were implemented to ensure the results were not influenced by confounding variables. A clearer description of the control measures would improve the interpretability of the results.**

**Response：**Thank you very much for your helpful comment regarding the lack of detail on control conditions in Experiment 1. In response, we have added a paragraph (see Page 14, Lines 291-300) at the end of the *Procedure* section to clearly describe the measures taken to control for potential confounding variables. Specifically, we now include information on stimulus standardization, Latin square design for target number sequences, randomization of response option positions, consistent use of verbal instructions, experimenter training, and the use of a structured practice phase. These measures were implemented to improve the interpretability of the results. We hope this revision addresses your concern.

**Revised Text in Manuscript:** “To control for potential confounding variables and enhance the internal validity of the experiment, several measures were implemented. All visual stimuli—including digits and dot arrays—were standardized in font type, size, color, and spatial layout across trials to eliminate perceptual biases. The sequence of target numbers was fixed based on the first two lines of a Latin square design to reduce order effects. In the tasks involving six alternative response options, the position of the alternatives was randomly assigned to prevent positional bias. Additionally, a standardized procedure was followed, with all participants receiving identical verbal instructions administered by a trained experimenter. A structured practice phase with feedback was also included to ensure participants understood the task requirements before proceeding to the formal testing phase.”

**5. Participant Selection Rationale:**
**While the authors state that they aimed to match the socioeconomic status of participants in Experiment 2 to those in Experiment 1, no details are provided on how this was assessed or confirmed. The different geographical location (Liangshan Prefecture vs. Dangwu Town and Mengyang Town in Experiment 1) raises questions about potential regional differences.**

**Response：**Thank you for your precious comments and advice. In response to your concern about the socioeconomic status of participants, we have provided additional details in the manuscript regarding how we ensured comparability between Experiments 1 and 2. The specific information regarding the preschools, tuition fees, and parental occupations can be found in the revised version of the manuscript (Page 21, Lines 454-459).

**Revised Text in Manuscript:** “All participating preschools were inclusive, non-profit institutions, with standardized tuition fees set by local government regulations, ranging from CNY 1,600 to 2,000 per semester. These schools served children from local families and did not apply any selective admission policies. Informal information obtained at the school level further suggested that most parents shared similar occupational backgrounds, primarily in small business, service industries, or skilled labor.”

**6. Time Gap Between Experiments:**
**Experiment 1 was conducted from April to May 2023, while Experiment 2 was conducted from December 2023 to January 2024. The potential impact of this 7–8-month gap is not addressed in the methodology. Please consider discussing whether this gap might have introduced seasonal or contextual changes that could affect participants’ behaviors or experiences. If the gap is not considered impactful, a justification for this assumption would strengthen the methodology.**

**Response：**Thank you for your insightful comment. We acknowledge that Experiments 1 and 2 were conducted in different months, with a time gap of 7–8 months between them. However, both experiments involved children of similar ages and targeted foundational numerical cognition, which is generally stable and less susceptible to seasonal or environmental changes (e.g., Dehaene, 1992). Given these considerations, we believe that the time gap between the two experiments is unlikely to have introduced any significant bias. We have updated the manuscript to clarify this point (Page 22, Lines 464-469).

**Revised Text in Manuscript:** “The experiment was conducted from December 11th, 2023, to January 4th, 2024, while Experiment 1 was carried out from April to May 2023. Although the two experiments were conducted in different months, both involved children of similar ages and focused on foundational numerical cognition [2], which is generally stable and less influenced by seasonal or environmental changes. Given this, we believe the time gap between the two experiments is unlikely to have introduced any significant bias.”

2. Dehaene, S. (1992). Varieties of numerical abilities. *Cognition*, *44*(1-2), 1-42.

**7. Sample Representation and Generalizability:
The participants were recruited from five preschools in two cities in China. While this is a reasonable geographical spread, it would be useful to comment on the demographic characteristics of the children. This would help assess the generalizability of the findings beyond this specific sample. The homogeneity of the sample should also be discussed to evaluate the external validity of the study's conclusions.**

**Response：**Thank you for your insightful comment. We agree that providing more details on the demographic characteristics of the participants is important for evaluating the generalizability of our findings. The children in our study were recruited from five preschools in two cities in China, and the sample was relatively homogeneous in terms of socioeconomic status, as all participating preschools were inclusive, non-profit institutions with similar tuition fees. Informal information from the schools indicated that most parents worked in small businesses, service industries, or skilled labor.

First, we have already expanded the demographic information in the *Methods* section in Experiment 2 (Page 21, Lines 454-459) while answering the fifth question. Second, we have addressed these points in the Limitations section (Page 32, Lines 700-705), where we acknowledge that the sample may not fully represent children from other regions or socioeconomic backgrounds beyond this local context. We suggest that the findings may be more generalizable to similar populations within China and emphasize that further research with more diverse samples would be valuable for assessing the broader applicability of the results.

**Revised Text in Manuscript:** “First, the participants were recruited from three towns across two provinces, which may limit the generalizability of the findings. The study also employed a cross-sectional design with a fixed task order, potentially introducing order effects and individual variability. Future research should consider broader sampling, longitudinal tracking, and fully randomized within-subject designs to enhance generalizability and experimental control.”

**8. Reminding Children to Count:
The inclusion of the "reminding children to count" condition in Experiment 2 is an interesting and relevant manipulation. However, more details on how the counting reminder was delivered separately and its duration would be helpful for evaluating the effectiveness of this intervention. Was there a standardized method for reminding the children to count, and how was it ensured that the reminder was consistent across participants?**

**Response：**Thank you for your insightful comment. In response, we have added further details to the *Procedure* section of Experiment 2 (see Page 22, Lines 471-477) to clarify how the counting reminder was delivered. Specifically, the reminder was standardized across all dot-related tasks and participants: the experimenter instructed each child to use their right index finger to point at the dots one by one while verbally counting them before responding. This consistent procedure ensured that the reminder was delivered uniformly across sessions and participants.

**Revised Text in Manuscript:** “Based on Experiment 1, Experiment 2 introduced a condition to remind children to count. In the AN, NA, AD, and DA tasks, the experimenter gave a standardized instruction before the child responded, saying, “Please count using your hand. How many dots are there?” To ensure consistency across participants, the experimenter instructed each child to use their right index finger to point at the dots one by one while verbally counting them aloud. This procedure was strictly followed to standardize the counting reminder across all children and trials.”

**Reviewer 2**

**1‌. Introduction Section‌. The current introduction lacks sufficient elaboration on real-world problems and research background.**

**Response：**Thank you for your valuable feedback. In the revised manuscript, we have significantly reorganized and expanded the *Introduction* section to enhance its clarity and logical flow. Specifically, we have added clear subheadings. These changes help to better elaborate on the real-world problems, theoretical background, and research motivations relevant to our study. We believe that the revised structure provides a more comprehensive and coherent context for readers and addresses the concern raised. We sincerely hope that the improvements meet your expectations.

**2‌. Literature Review‌. It is recommended to add a dedicated "Literature Review" section. Reorganize existing content from the Introduction to systematically summarize prior studies with logical taxonomies.**

**Response：**Thank you for your helpful suggestion. In the revised manuscript, we have created a dedicated Literature Review section and reorganized the relevant content that was previously embedded in the *Introduction*, systematically summarizing prior research with a clearer logical structure. Specifically, we have added clear subheadings, including *Mapping Direction*, *Developmental Order of Numerical Mapping*, *Mapping Differences by Number Size*, *Effect of Counting on Numerical Mapping*, and *The Current Study*. We believe these changes enhance the clarity, coherence, and accessibility of the background information. We appreciate your guidance in helping us improve the manuscript.

3. **Reference Timeliness‌. Add some relevant studies within five years.**

**Response：**Thank you for your valuable suggestion. In the revised manuscript, we have carefully reviewed and incorporated additional recent literature. Specifically, we added 20 important references published within the past five years. As a result, the manuscript now includes 31 references from the past five years out of a total of 71 references, accounting for 43.66%. We believe that these updates enrich the manuscript and ensure that it reflects the latest developments in the field. We sincerely appreciate your feedback, which has helped us strengthen the quality and relevance of the paper.

4. **Comparative Analysis of Experiments‌. The comparative analysis between the two experiments is insufficient.**

**Response：**Thank you for your insightful comment. In the revised manuscript, we have strengthened the comparative analysis between Experiment 1 and Experiment 2. Specifically, Experiment 2 was designed based on Experiment 1 by adding a “counting reminder” condition, aiming to further explore how reminding children to count influences the evaluation of the developmental order and the symmetry of mapping direction in numerical mappings.

In the *Results and Discussion* and *General Discussion* sections, we systematically compared the two experiments from three perspectives: (1) the symmetry of mapping directions, (2) the developmental level of numerical mapping, and (3) the developmental order of mappings. Comparative analyses were conducted separately for each age group (3-year-olds and 4-year-olds), and within each age group, we further distinguished between small and large number ranges.

The findings show that reminding children to count could improve their performance on array–number word and array–digit mappings, leading to changes in both the symmetry of mapping directions and the developmental order of mapping paths, which was consistent with our hypotheses. In conclusion, we emphasized that number size and counting reminders play significant roles in shaping researchers’ assessments of the developmental order of numerical mapping in preschool children.

We sincerely thank you for your constructive feedback, which helped us improve the rigor and depth of our manuscript.

**5.‌** **Innovation Clarification. The articulation of research novelty remains vague.**

**Response：**Thank you for your valuable comment. We have clarified the innovations of our study in the manuscript (see Pages 31-32, Lines 679-689). Specifically, based on previous debates, we examined the symmetry of mapping direction and the developmental order of mapping paths from the perspectives of number size and the effect of reminding children to count. We found that these two factors influence the assessment of the developmental order of numerical mapping, providing two pieces of empirical evidence that help explain discrepancies reported in earlier studies and offering a reference for the cultivation and assessment of preschool children's numerical mapping ability. In particular, we observed that the developmental orders differed between small- and large-number mappings, supporting the existence of two distinct underlying mechanisms: the object tracking system (OTS) and the approximate number system (ANS). Moreover, we identified that the critical period for the emergence of numerical mapping abilities occurs around the age of two, offering further insights into early educational interventions. These findings contribute novel perspectives to the field of early numerical cognition.

**Reviewer 3**

**1. Why classify the mapping paths into three rather than six?**

**Response：**Thank you for your thoughtful comment. We appreciate your attention to the classification of mapping paths. In response, we would like to clarify that our approach follows the analytical method adopted by Benoit et al. (2013). As they stated, “*These tests confirmed that there were no reliable differences between the mapping directions of the representation pairs. Consequently, in subsequent analyses, we collapsed the two tasks involving the same representation pairs.*” Similarly, in our Experiment 1, we conducted Wilcoxon signed-rank tests to examine the differences between the two directions of each representation pair, and the results revealed no reliable differences overall (see Tables 1–3). Given this pattern, we followed the precedent set by Benoit et al. (2013) and collapsed the mapping directions into three broader categories (i.e., the three numerical mapping paths). This approach both reflects the empirical findings in our data and facilitates direct comparison with previous work. We have added a sentence in the *Results and Discussion* section of Experiment 1 (Page 15 Lines 310-312) to clarify this rationale.

**Revised Text in Manuscript:** “Therefore, to maintain consistency with Benoit et al.’s (2013) analysis and to facilitate comparability across studies, we also collapsed across mapping directions and analyzed only the three primary mapping paths in subsequent analyses.”

**2. What theoretical and empirical evidence supports the developmental order of numerical mapping? Are there studies indicating that children follow a fixed sequence in acquiring numerical mapping skills, or could this sequence be influenced by factors such as culture, educational background, or task type?**

**Response：**Thank you for your thoughtful comment. The developmental order of numerical mapping has been shown to vary across studies, as different research has suggested different patterns. For example, Benoit et al. (2013) observed a developmental order of array–number word (path 2) → digit–array (path 3) → digit–number word (path 1). In contrast, Jiménez Lira et al. (2017) found a 1→2→3 order, while Hurst et al. (2017) and Marinova et al. (2021) suggested that children acquire paths 1 and 2 first, followed by path 3 (1 and 2 → 3). These differences highlight that the developmental order may depend on symbolic familiarity, task format, and cultural or educational background.

Our study found that both number size and counting reminders influenced mapping performance, suggesting that the developmental order of numerical mapping is context-dependent rather than universal. Specifically, the developmental order varies between small and large number ranges (see Table 7), supporting the dual-system theory of number processing. Small numbers are more likely to be processed through an object tracking system (OTS), while large numbers engage the approximate number system (ANS). We have elaborated on these findings in the *Results and Discussion* sections of Experiments 1 and 2, as well as in the *General Discussion* section of the revised manuscript.

**3. The relationship between mapping direction and developmental order: If different mapping directions exist, does the developmental path remain consistent? For instance, do array-to-digit and digit-to-array mappings develop simultaneously, or is there a sequential progression?**

**Response：**We sincerely thank you for raising this important and thought-provoking question regarding the relationship between mapping direction and developmental order. In our view, the performance of children in different mapping directions (e.g., array-to-digit vs. digit-to-array) is fundamentally symmetric at the representational and cognitive level. Although asymmetries were observed in Experiment 2 when children were reminded to count, we argue that these differences are task-induced, rather than reflecting an inherent developmental order between mapping directions.

First, the results of Experiment 1 in the present study, where children were not reminded to count, showed no significant differences in mapping direction across the three mapping paths. This symmetric performance pattern aligns with Benoit et al. (2013), who also reported no significant directional differences under similar conditions. These findings suggest that children’s numerical mapping abilities do not inherently favor one direction over another.

Moreover, in Experiment 2, the asymmetry in mapping direction emerged only among 3-year-olds, and only on paths 2 and 3 in the large number range, when children were reminded to count. Importantly, this asymmetry was no longer statistically significant among 4-year-olds (only marginally significant), indicating that the asymmetry may reflect a temporary effect tied to task difficulty and age-related processing capacity, rather than a stable developmental progression. Furthermore, mapping direction remained symmetric for path 1 and for paths 2 and 3 in the small number range, reinforcing the notion that mapping direction itself is not fundamentally asymmetric in early development.

We believe these findings are best explained by task-related factors. In the NA and DA tasks, children must select the correct target from six candidate dot arrays, which increases perceptual and working memory demands. In contrast, AN and AD tasks require judging only a single array, making them less cognitively taxing. This interpretation is supported by previous research. For instance, Hurst et al. (2017) found that children performed slightly better on AN than NA tasks and counted far more often in AN trials (56%) than in NA trials (9%), attributing the difference to the cognitive demands of evaluating multiple arrays. Similarly, Jiménez Lira et al. (2017) reported that children performed better in two-alternative forced choice tasks (e.g., NA, DA) than in direct array identification tasks (e.g., AN), highlighting the role of task format.

Taken together, these findings suggest that the asymmetries in direction observed in limited conditions are task- and age-related, and do not reflect a consistent or inherent developmental order of directional mappings. Therefore, we argue that it is both theoretically and methodologically sound to collapse mapping direction into broader “mapping paths”, which better capture the nature of representational transformations without being confounded by task complexity. We greatly appreciate your comment, which has helped us to clarify and strengthen the theoretical framework and empirical interpretation of our study.

**Reviewer 4**

**Introduction
Major Concerns**

**1. Review of Previous Research for Replication (Lines 28–37, 115–119)
The introduction briefly mentions previous studies with differing findings to justify replication. However, a detailed comparison of these studies is necessary to identify potential sources of discrepancies. Otherwise, differences in findings may simply reflect variations in experimental design rather than a meaningful replication attempt.**
**• The summary suggests that differences in experimental materials (e.g., number size), procedures, or participant demographics (e.g., socioeconomic background, education levels) may contribute to variations in findings. However, these factors are not sufficiently reviewed.**

**• How do these previous studies compare in terms of similarities and differences? What aspects might have led to different outcomes?**

**Response：**We sincerely thank you for pointing out the need for a more detailed comparison of prior studies to clarify the sources of discrepancies in findings. We have now revised the *Introduction* to provide a more systematic comparison and explicitly highlight potential sources of variation across studies, as requested.

In our revised version, we reviewed and compared representative studies on numerical mapping in early childhood. While these studies share a common goal of investigating the developmental order and directionality of numerical mappings, several methodological differences may have contributed to divergent results.

1) Experimental procedures (counting instructions):
   One crucial factor is whether children were reminded to count. In our Experiment 2, children were explicitly encouraged to count the arrays, while in Benoit et al. (2013), no explicit counting instructions were provided. Hurst et al. (2017) noted that “counting was neither prevented nor encouraged on any task,” and found that children spontaneously counted more frequently in the AN task (56%) than in the NA task (9%), likely due to differences in task complexity (i.e., single vs. multiple arrays). These findings support our interpretation that performance differences across mapping directions may be task-induced rather than reflecting inherent cognitive asymmetry.
2) Number size:
   Most studies—including ours—reported better performance for small numbers than for large numbers. However, many previous studies did not distinguish number size when comparing developmental orders of mapping paths. For instance, Benoit et al. (2013) and Hurst et al. (2017) analyzed developmental patterns across all number ranges combined, without separating small and large numbers. In contrast, our study explicitly differentiated between number sizes and revealed that the developmental order of mapping paths differed between small and large numbers.
3) Participants’ characteristics:
   Although not always explicitly reported, differences in children's age range, counting skills, and sociocultural or educational backgrounds may have influenced mapping performance. For example, Jiménez Lira et al. (2017) tested children from a Canadian city, Hurst et al. (2017) examined American children, and Benoit et al. (2013) studied French children, with their findings showing notable differences. Our participants were recruited from Chinese kindergartens, providing new cross-cultural data that may help clarify developmental trends.
4) Task format and difficulty:
   Studies vary in task design, including whether they required direct mapping, forced-choice responses, or verbal explanations. Jiménez Lira et al. (2017) found that the two-alternative forced-choice task was easier than the direct quantity naming task, whereas Benoit et al. (2013) reported that the six-alternative forced-choice task and the direct naming task were comparable in difficulty. Consistent with Benoit et al. (2013), our study also found no significant difference in difficulty between the six-alternative forced-choice task and the direct naming task.

Taken together, these differences suggest that observed discrepancies in previous findings may stem from differences in task structure, stimulus properties (e.g., number size), and experimental instructions (e.g., whether to encourage counting), rather than reflecting inconsistencies in developmental patterns themselves.

We have substantially reorganized and expanded the *Introduction* to improve its clarity and logical flow. Specifically, we introduced clear subheadings: *Mapping Direction*, *Developmental Order of Numerical Mapping*, *Mapping Differences by Number Size*, *Effect of Counting on Numerical Mapping*, and *The Current Study*. These additions allow us to more systematically present the real-world issues, theoretical background, and research motivations underlying our work. We believe that the revised structure provides a more comprehensive and coherent framework for readers and fully addresses the concerns raised. We sincerely hope that these improvements meet your expectations.

**• Based on this review, what specific experimental design choices were made in the current study to ensure replication?**

**• The manuscript states that it closely follows Benoit et al. (2013) in methodology. If so, why was this particular study chosen?** **Were there any modifications to the methodology?**

**Response：**We sincerely thank you for the thoughtful questions regarding our study’s methodology and its alignment with previous research.

We chose Benoit et al. (2013) as a reference study for several reasons. First, it is a well-established study that offers a comprehensive examination of the developmental order of number mapping paths in young children, covering all three major paths (digit-to-array, number word-to-array, and digit-to-number word). Second, Benoit et al. (2013) demonstrated an important result: children showed superior performance in mapping path 3 (digit-to-array mapping) compared to other paths, a finding that contrasts with other studies. This unique aspect of their results made it an ideal choice for comparison, as we sought to explore and validate their findings further in a different context. Third, Benoit et al. (2013) did not differentiate between small (1-3) and large (4-6) number ranges when exploring the developmental order of number mapping. This lack of distinction provided a valuable clue for improving our methodology, as we hypothesize that small and large numbers may follow distinct developmental patterns. Given that Benoit et al. (2013) has had a significant impact on the field, it was a logical choice for our comparison.

Building on Benoit et al. (2013), we introduced two key modifications in our study. Firstly, we separately assessed small (1-3) and large (4-6) number ranges, whereas Benoit et al. (2013) combined small and large numbers when evaluating the developmental order of number mapping paths. We hypothesize that small and large numbers follow distinct developmental patterns, which is why we believe distinguishing these two ranges allows for a more nuanced understanding of developmental progression. By separately examining these ranges, we aim to refine the understanding of how number size influences the acquisition of different mapping paths. Secondly, in contrast to Benoit et al. (2013), where no explicit counting reminders were given, we introduced a counting reminder condition in Experiment 2. Based on prior research indicating that counting can enhance children’s performance on numerical tasks, particularly for larger numbers that are less easily processed without counting, we hypothesized that reminding children to count might improve their ability to map symbolic and non-symbolic numbers. This modification was made to explore whether counting could serve as a valuable tool in improving children’s mapping abilities, particularly for larger numbers where estimation alone may be insufficient.

Thus, while our study closely follows Benoit et al. (2013) in terms of its core methodology and experimental design, these modifications—separating small and large number ranges and introducing the counting reminder—allow us to address gaps in the original study and expand our understanding of the developmental order of number mapping.

**2. Review of Previous Research on the Developmental Order of Numerical Mapping (Lines 38–75)
Some explanations in the introduction appear inconsistent with reported findings.
• Benoit et al. (2013) is cited as explaining why children learn Path 1 before Path 3, yet their findings indicate a developmental sequence of 2 → 3 → 1. Could this discrepancy be clarified?
• Hurst et al. (2017) presents two accounts:
o Quantity account: 2 → 3 → 1
o Symbolic account: 2 → 1 → 3
However, their findings indicate 2 & 1 → 3, which contradicts both accounts. Could this be addressed?**

**• Additionally, why does the manuscript reference Hurst et al. (2017) rather than Benoit et al. (2013) to explain developmental order, despite closely following Benoit et al. (2013) in methodology? Would it be more appropriate to reference Benoit et al. (2013)’s explanations instead? If not, are there limitations in Benoit et al.’s explanation that justified focusing on Hurst et al. (2017)?**

**Response：**Thank you for these thoughtful and constructive comments. We are grateful for the opportunity to clarify our use of prior literature in describing the developmental order of numerical mappings.

(1) Clarifying the discrepancy between cited theoretical explanations and empirical findings

In our manuscript, the explanations cited from Benoit et al. (2013) and Hurst et al. (2017) in Lines 38–75 were based on the theoretical expectations outlined in the introductions of those studies, rather than their interpretations of the empirical results.

For instance, Benoit et al. (2013) initially hypothesized that children would first acquire the digit-to-word mapping (path 1) before the digit-to-array mapping (path 3), due to the symbolic similarity and frequent exposure to number words being read aloud. However, their findings indicated a developmental order of 2 → 3 → 1, and in their discussion, they revised their interpretation, suggesting that digit-to-array mappings may be easier due to the permanence of the visual representations and lower working memory demands.

Similarly, Hurst et al. (2017) proposed two competing theoretical accounts: the quantity account predicted 2 → 3 → 1; the symbolic account predicted 2 → 1 → 3. Yet, their actual findings showed better performance on paths 1 and 2 compared to path 3, suggesting that path 3 is acquired later. In the discussion, they proposed that children may first acquire symbolic-symbolic and symbolic-quantity mappings and only later infer quantity-symbolic mappings (path 3) via an indirect route. We have already revised the *Introduction* to make these distinctions more explicit and clarify the relationship between the theoretical accounts and empirical results in both studies (see Pages 5-6, Lines 81–108).

(2) Why Hurst et al. (2017) was used to explain developmental order

Although our methodological design is more closely aligned with Benoit et al. (2013), we referenced Hurst et al. (2017) in the explanation of developmental order for two reasons:

1) Hurst et al. (2017) explicitly compared multiple theoretical accounts (quantity-based vs. symbolic mediation) and discussed their explanatory power in light of their findings. This comparative framework provided a clearer structure for introducing competing hypotheses about mapping order.
2) Benoit et al. (2013) did not explicitly discuss the symbolic mediation route or present contrasting hypotheses in the same structured way. While their discussion provided important post hoc explanations, they were less explicitly framed as alternative developmental accounts.

Nevertheless, we agree that it would be more balanced to also reference Benoit et al.’s discussion-based explanation, especially since our study closely follows their methodology. In our revision, we have already integrated both studies’ interpretations more clearly—highlighting Benoit et al. (2013)’s post hoc rationale as well as Hurst et al. (2017)’s account comparisons—so that readers can better understand both the theoretical context and methodological grounding of our work.

**•** **Finally, the second hypothesis is quite general. Could the predicted outcomes be specified in more detail? Given that Path 3 is relatively robust in prior research (except in Benoit et al. (2013)), further elaboration is needed.**

**Response：**Thank you for your valuable comment. In response, we have revised Hypothesis 2 to specify the predicted outcomes in more detail (see Page 10, Lines 196–203). Specifically, under the condition without counting reminders, we now hypothesize that for small numbers (1–3), path 2 (array/number word) will show the best performance, while for large numbers (4–6), path 1 (digit/number word) will show the best performance. These predictions have been added to the revised manuscript to clarify the expected developmental patterns across numerical ranges.

**Revised Text in Manuscript:** “(2) the developmental order of numerical mapping paths would differ between small and large numbers—for example, under the condition without counting reminders, we expect the best performance on path 2 (array/number word) for small numbers (1–3), and the best performance on path 1 (digit/number word) for large numbers (4–6); and (3) reminding children to count would improve their performance on paths 2 and 3 for large numbers, which require counting, thereby altering the symmetry of mapping directions and the developmental order of mapping paths.”

**Methodology**
**Major Concerns
1. Forward and Backward Directions (Lines 159–185)
o In Figure 1, Path 1 (DN) appears to be a ‘recall’ task with no alternatives, while ND is a ‘recognition’ task with six-alternative forced choices. Are these forward (DN) and backward (ND) directions truly equivalent?
o A more comparable approach might be:
♣ DN: Seeing the number “three” briefly and hearing six audio alternatives sequentially, then selecting the correct one.
♣ ND: Hearing the word "three" briefly and seeing six visual alternatives sequentially, then selecting the correct one.
Could this be a limitation of the design?**
**Response：**Thank you for your thoughtful observation. We agree that the DN and ND tasks differ in response format—DN being a recall task without alternatives, and ND a recognition task with six forced-choice options. This asymmetry is indeed a limitation of our current design. We intentionally adopted this structure to align with the methodology of Benoit et al. (2013), which serves as a key reference point for our study. Maintaining methodological consistency allows for meaningful comparison of results across studies. We acknowledge this limitation in the revised manuscript and will consider incorporating more symmetric task formats in future research to address this issue (see Pages 32-33, Lines 710–713).

**Revised Text in Manuscript:** “Third, the asymmetry in forward and backward mapping tasks involving number words may have introduced differences in cognitive demands. Although this design aligned with prior studies (e.g., Benoit et al., 2013), future research should adopt more balanced task structures.”

**Minor Concerns
2. Stimuli Presentation (Lines 143–146)
The manuscript states that both laptops and printed versions were used. If printed versions are more engaging for children, this could introduce performance differences.
o Which version was used for which task? Were they switched during the experiment or research?
o How many participants used only laptops vs. only printed versions?**

**Response：**Thank you for your thoughtful comment. We apologize for the lack of clarity in the original text. To clarify, all participants were presented with the printed version of the stimuli throughout the study. The mention of laptop-based stimuli referred only to the design and spatial layout of the materials, which were adapted from Benoit et al. (2013) to ensure comparability.

We chose to use printed materials because they offer a more developmentally appropriate and child-friendly mode of interaction for young children. Compared to screen-based formats, printed materials allow for more intuitive engagement and help minimize distractions from digital interfaces. This choice also aligns with previous studies involving preschool-aged participants (Jiménez Lira et al., 2017). The manuscript has been revised accordingly to reflect this clarification (see Page 11, Lines 225–233).

**Revised Text in Manuscript:** “The experimental materials were presented in a printed format for all participants, as printed materials offer a more developmentally appropriate and child-friendly mode of interaction for young children. Compared to screen-based formats, printed materials allow for more intuitive engagement and reduce potential distractions related to digital interfaces. The design and spatial layout of the stimuli (including dot arrays and digits) were adapted from Benoit et al. (2013) [5], who used laptop-based presentations. While the mode of presentation differed, we maintained the same core features in stimulus design to ensure comparability. This approach also aligns with previous research using printed stimuli with preschool-aged children [8].”

**3. Handling of Participants Scoring Zero in the Practice Phase (Lines 197–198)
o If children who scored zero still understood the instructions, this should be explicitly stated.
o Including data from participants who did not understand the task may not be appropriate.**

**Response：**Thank you for your insightful comment. Our intention in including all 2-year-old participants in the test phase—regardless of their practice phase performance—was to capture a comprehensive picture of early numerical mapping development, including those at the very initial stages. However, we acknowledge that it was not possible to objectively determine whether children who scored zero truly understood the task, given their developmental stage and limited verbal feedback. To address this potential concern, we conducted a separate analysis excluding children who scored zero across all test conditions. This allowed us to focus on the subset of children who likely had at least a minimal understanding of the task. As reported in the manuscript, the results of this analysis (using the Wilcoxon test) showed that these children performed significantly above chance on mapping paths 1 and 2 for small numbers, providing further support for the emergence of mapping ability at this age.

**Results and Discussion**
**Major Concerns
1. Excessive Use of Statistical Tests and Lack of p-Value Correction
o Given the number of statistical tests, p-value corrections (e.g., Bonferroni) should be considered to minimize Type I errors.
o If the study follows previous research in not applying p-value corrections, this should be clearly stated. However, without corrections, the increased risk of Type I errors could lead to misleading interpretations.**

**Response：**Thank you for your valuable suggestion. Although the reference study by Benoit et al. (2013) did not report *p*-value corrections, we agree that it is important to control for Type I errors when conducting multiple comparisons. Therefore, we have now applied Bonferroni corrections for multiple comparisons and revised the relevant sections accordingly in the manuscript (see Page 17, Lines 349–354; Pages 19-20, Lines 405–436; Pages 25-26, Lines 523–556). Importantly, after applying the corrections, the main experimental results and conclusions remained largely unchanged. We appreciate your thoughtful feedback, which has helped us enhance the rigor and reliability of our statistical analyses.

**2. Conflicting Arguments (Lines 224–234)
o The claim that alternative forced-choice designs influence results is not supported, as no significant differences were observed in any path in the current study, which is the argument that selecting from six alternatives (NA) is equivalent to determining the number in an array (AN).**

**Response：**We appreciate your careful reading and valuable comment. We would like to clarify that we did not intend to claim that selecting from six alternatives (NA) is more difficult than determining the number of items in an array (AN). Rather, our interpretation was that the six-alternative forced-choice design used in the NA mapping task increased the task’s complexity to the extent that it was comparable in difficulty to the AN mapping task, which required children to determine the quantity of a dot array without alternatives. This design choice may help explain why children showed no significant differences between NA and AN mapping in the current study, as both tasks posed comparable cognitive demands. To avoid any misunderstanding, we have revised the relevant section of the manuscript to more clearly convey this interpretation (see Page 16, Lines 331–336).

**Revised Text in Manuscript:** “In this study, children were asked to choose one of six candidate arrays in the NA mapping [5], which increased the cognitive demands of the task (e.g., visual scanning and comparison). This elevated difficulty may have made the NA task comparable in challenge to the AN task, where children needed to directly determine the number represented by a single dot array. This design might explain the lack of a significant difference between NA and AN mapping performance observed in our results.”

**3. Indirect Comparisons (Lines 265–271)
o The argument that one condition is below chance level, while another is above chance level, therefore one is superior is weak. A direct comparison is necessary to support this claim, especially since it involves ethnicity.**

**Response：**Thank you for this important observation. We agree that making claims about group differences based solely on significance versus non-significance can be misleading, especially when the comparison involves samples from different cultural backgrounds. To avoid overinterpretation and ensure scientific rigor, we have removed the statement suggesting that Chinese children outperformed French children. Instead, we now neutrally describe the observed discrepancies between the studies and emphasize that these differences should be interpreted with caution (see Page 18, Lines 372–376).

**Revised Text in Manuscript:** “These results differ from those reported by Benoit et al. (2013), in which 3-year-old children’s performance on most mapping paths was below chance. While both studies used similar materials and procedures, the performance differences may reflect variability in children’s numerical development across samples or differences in educational or cultural context.”

**4. Chance Level Analysis (Lines 259-264, 281–287)
o The assumption that the expected chance level is 1/6 may not be appropriate for all conditions. Could this be reconsidered, particularly for conditions involving number words as outputs?
o Excluding zero scores may be problematic, as zero values still carry meaning in random selection probabilities.**

**Response：**Thank you for this important comment. In our study, we first conducted a primary analysis including all participants, as shown in Table 4. This analysis revealed that nearly half (47.5%) of the 2-year-old children scored zero on all mapping tasks. Zero scores are indeed meaningful and were fully considered in this initial analysis. However, to further explore whether any 2-year-old children had begun to demonstrate emerging mapping abilities, we conducted an additional analysis excluding those who scored zero across all tasks. This supplementary analysis, shown in Table 5, aimed to reveal early signs of mapping competence among children who responded at least minimally.

We have now clearly clarified this two-step approach in the manuscript (see Pages 18-19, Lines 386–396). This dual analysis allows us to balance a comprehensive view of all participants with a focused exploration of children showing initial task engagement.

**Revised Text in Manuscript:** “For 2-year-old children, their mapping performance was not significantly above chance in any conditions (either below or not significantly above the chance level). The main reason for this was that 19 out of 40 2-year-old children (47.5%) scored zero on all mapping tasks. Therefore, to master the mapping performance of 2-year-old children who have started to develop mapping abilities, we adopted a two-step approach in the analysis. First, we included all participants, including those who scored zero on all tasks (see Table 4). Then, we conducted a supplementary analysis excluding children with zero scores, as this allowed us to further explore the performance of children who demonstrated mapping abilities (see Table 5). This second analysis was performed using the Wilcoxon test. The results showed that children’s mapping performance was above the chance level on paths 1 and 2 for small numbers.”

**5. Counting and Mapping Performance (Line 387-388)
o In Figure 3, four out of eight conditions (or three out of four in large numbers) showed significant biases, indicating partial improvement in mapping performance rather than a robust effect.**

**Response：**Thank you for your thoughtful comment regarding the robustness of the reminder effect on mapping performance. We appreciate your observation that, in Figure 3, significant improvements were observed in only part of the conditions, which may suggest a partial rather than a robust effect.

In response, we would like to clarify that the effect of counting reminders varies across age groups and number ranges due to differences in task difficulty and baseline performance levels:

- For 3-year-old children, path 3 appears to be more cognitively demanding. As such, even a marginal improvement (Z = -1.793, *p* = 0.073) in the small number range under the reminder condition suggests that reminders may help children cope with the task’s inherent difficulty.
- For 4-year-old children, performance on path 2 and path 3 within the small number range approached ceiling levels, with high accuracy regardless of the reminder condition. This ceiling effect likely limited the observable impact of reminders in these conditions. In contrast, within the large number range, where the task remained more challenging, significant improvements were indeed observed on both path 2 and path 3 when reminders were provided.

We have added a clarification of this point in the revised manuscript (Page 24, Lines 508–521) to more accurately interpret the effect of counting reminders in light of age-related differences and potential ceiling effects.

**Revised Text in Manuscript:** “For 3-year-old children, the results of the Mann–Whitney U test indicated that performance on path 2 was significantly better with reminders than without reminders, regardless of whether the numbers were small (Z = -2.454, *p* < 0.05) or large (Z = -3.373, *p* < 0.01). In addition, performance on path 3 for small numbers was slightly better with reminders than without reminders (Z = -1.793, *p* = 0.073), suggesting a trend toward improvement despite the high cognitive demand of this task at age 3. For 4-year-old children, performance on paths 2 and 3 for large numbers was significantly better when they were reminded to count than when they were not (path 2: Z = -3.070, *p* < 0.01; path 3: Z = -2.234, *p* < 0.05). However, for small numbers, performance on both paths 2 and 3 was already close to ceiling levels, which may have limited the observable effects of reminders in these conditions. Therefore, it can be concluded that in numerical mapping tasks, reminding children to count can improve their mapping performance on paths 2 and 3, especially when the tasks are relatively difficult and children's baseline performance leaves more room for improvement.”

**6. Potential Alternative Explanations and Counterarguments
o The study suggests that reminding children to count (using a single hand) improves their performance. However, alternative explanations should be discussed.
o For example, one key factor may be working memory. Since performance improves with age even in the absence of explicit counting (Lines 242–250), the explicit counting alone may not be the sole driving factor. Instead, working memory development could facilitate mental counting (arithmetic) without external aids. In this case, children may already does engage in counting (mental arithmetic), and explicitly counting with a single hand—potentially serving as a visible extension of working memory—could further enhance performance.
o If a full discussion is not feasible, reconsider at least acknowledging alternative explanations in the limitations section.**
**Response：**Thank you very much for this insightful suggestion. We agree that working memory development is an important factor in children’s numerical cognition. However, we would like to clarify that in our study design, the effect of counting reminders was analyzed within each age group, by comparing children’s performance with and without reminders under the same age condition. Therefore, the observed differences in performance are unlikely to be attributable to age-related improvements in working memory alone.

In our study, the “counting” prompted by the reminder was an explicit and concrete behavior, where children were instructed to use their fingers to point to each dot in the array while verbally counting. This kind of physical, one-to-one correspondence strategy supports early numerical understanding and differs from internalized mental arithmetic. That said, we acknowledge that finger-based counting may serve as a visible scaffold that interacts with children’s existing cognitive capacities, including working memory. We have included this possible interpretation as a supplementary explanation in the *Limitations and Future Directions* section (Page 33, Lines 713–715), noting that future research could further explore how individual differences in cognitive development may influence the effectiveness of external counting aids.

We appreciate this comment, which allowed us to clarify our design and better articulate the potential mechanisms underlying the observed effects.

**Revised Text in Manuscript:** “Additionally, although the effects of counting reminders were analyzed within each age group, counting strategies may still interact with cognitive resources such as working memory, warranting further investigation.”

**Minor Concerns
7. Performance Collapsing Across Directions
o Did the study collapse performance across the two mapping directions for analyses other than investigating mapping directions? This seems likely, given that Table 1 shows no differences, but this should be explicitly stated.**
**Response：**Thank you for your careful reading and helpful comment. Yes, for analyses other than the investigation of mapping direction effects, we collapsed performance across the two directions. This was done to maintain consistency with the analytic approach used in Benoit et al. (2013) and to facilitate cross-study comparisons. We have provided an explanation in the manuscript (see Page 15, Lines 310–312). We appreciate your suggestion and have now made this point clearer in the relevant section of the manuscript.

**Revised Text in Manuscript:** “Therefore, to maintain consistency with Benoit et al.’s (2013) analysis and to facilitate comparability across studies, we also collapsed across mapping directions and analyzed only the three primary mapping paths in subsequent analyses.”

**8. Figures and Tables
o Revise the figures and tables to ensure they align with the statistical methods used. Specifically, check whether the current study compares means between groups (Student t-test), compares distributions of independent groups (Mann–Whitney U test), or tests whether the median of paired differences significantly differs from zero (Wilcoxon signed-rank test). For the latter two methods, reconsider presenting statistics other than means, such as medians and interquartile ranges.**
**Response：**Thank you for your valuable suggestion. We have carefully considered the normality of the data and found that while the data deviates from normality, previous studies in the field, including those by Benoit et al. (2013) and Hurst et al. (2017), have reported results using means and standard deviations for similar non-parametric data. In line with these precedents, we have opted to retain the presentation of means and standard deviations in our study for consistency with the existing literature. Additionally, we have ensured that appropriate non-parametric statistical tests were used to analyze the data, and we believe this approach is justified given the specific context of our research. We hope this approach aligns with the overall goals of the study while maintaining comparability with previous work in the field.

**9. Clarification of Graphs and Tables
o Each graph and table should explicitly define terms such as "Path 1, 2, 3," "first/second direction," asterisk criteria, and abbreviations. Specifically, what do "first direction (M1)" and "second direction (M2)" represent? For example, does M1 correspond to DN and M2 to ND?**
**Response：**Thank you for your valuable suggestion. We have revised the captions for all figures and tables to clearly define the terms “path 1,” “path 2,” “path 3,” “M1,” and “M2.” For closely related tables presented together (e.g., Tables 1–3), we provided the full definitions in the caption of Table 1 and noted in Tables 2 and 3 that the terminology is consistent with Table 1. For all other figures and tables, we added explicit definitions in each caption to ensure clarity and readability for all readers. Thank you again for pointing this out.

**Revised Text in Manuscript:** “**Note:** Small numbers (1–3), large numbers (4–6). M1 refers to the first direction of each mapping path (e.g., DN, AN, DA), and M2 refers to the second direction (e.g., ND, NA, AD). Mapping path abbreviations are as follows: DN = digit-to-number word, ND = number word-to-digit, AN = array-to-number word, NA = number word-to-array, DA = digit-to-array, and AD = array-to-digit. The same definitions and notation apply to Tables 2 and 3.”

**10. Statistical Results (Line 249)**
**o The Mann-Whitney U test results appear to be missing. Are all results significant at p < .05, .01, or .001?**
**Response：**Thank you for your comment. We have now added the results of the multiple comparisons with Bonferroni correction to the manuscript and have clearly indicated the corresponding significance levels (p < .05, .01, or .001) where appropriate (see Page 15, Lines 349–354).

**Revised Text in Manuscript:** “Results of the multiple comparisons with Bonferroni correction revealed that the mean numerical mapping scores of 2-year-old children were significantly lower than those of 3-year-olds (path 1: *p* < 0.05; paths 2 and 3: *ps* < 0.001) and 4-year-olds (*ps* < 0.001). Additionally, 3-year-olds scored significantly lower than 4-year-olds across all paths (*ps* < 0.001). These findings indicated a significant age-related improvement in numerical mapping performance across all mapping paths [5,7-9].”

**11. p-Value Formatting
o In tables, p-values should not be reported as 0.000. Instead, the standard convention would be to report < .001.**
**Response：**Thank you for your careful observation. We have revised all relevant tables in the manuscript to replace p-values reported as 0.000 with the standard format "< 0.001" in accordance with academic conventions.

**12. Figure 4 Layout
o Graphs in Figure 4 should match the statistical analysis. If the focus is on the developmental order of mapping performance, then the graphs should be arranged accordingly for small and large numbers with appropriate asterisks.**

**Response：**We sincerely thank you for this insightful suggestion. While Figure 4 was initially designed to align with the layout of Benoit et al. (2013), we fully agree that reordering the graphs to reflect the developmental sequence of mapping performance—separately for small and large number ranges—will make the visual comparison more intuitive and better aligned with our analysis. In response, we have revised both Figure 2 and Figure 4 accordingly, adding significance markers to improve clarity and enhance the interpretation of developmental trends.

**Revised Figures in Manuscript:**

**Fig 2.** Mean number of correct responses for paths 1, 2, and 3, by number size.

*Note: path 1 (digit/number word), path 2 (array/number word), and path 3 (digit/array).*

**Fig 4.** Mean number of correct responses for mapping paths 1, 2, and 3 with reminding children to count, by number size

*Note: path 1 (digit/number word), path 2 (array/number word), and path 3 (digit/array).*

**Other Concerns
Major Concerns
• Data Availability
o The manuscript states that "all data are fully available without restriction", yet another section states that data is available upon reasonable request. This inconsistency should be clarified.**
**Response：**Thank you for pointing out this inconsistency. We have revised the manuscript to ensure the data availability statement is accurate and consistent throughout. To facilitate access, we have uploaded all stimuli, raw data, and related experimental materials to the Open Science Framework at https://osf.io/rs2up/, where they are now publicly available without restriction.

**Minor Concerns**
**• Citation formatting (e.g.,** **Benoit et al. (2013)) may need revision.**

**Response：**Thank you for your comment. We have carefully reviewed all in-text citations and the reference list to ensure consistency with APA formatting guidelines. The use of “Benoit et al. (2013)” and similar citations follows the APA style for references with three or more authors. We appreciate your attention to detail.

**Reviewer 5**

**1. The literature review is not sufficiently comprehensive. Studies from the past five years account for only 20% of the references, raising concerns that key advancements in this area may have already been established. It is recommended that more recent studies be incorporated to ensure the discussion reflects the current state of research and justifies the necessity of this study.**
**Response：**Thank you for your valuable suggestion. In the revised manuscript, we have carefully reviewed and incorporated additional recent literature. Specifically, we added 20 important references published within the past five years. As a result, the manuscript now includes 31 references from the past five years out of a total of 71 references, accounting for 43.66%. We believe that these updates enrich the manuscript and ensure that it reflects the latest developments in the field. We sincerely appreciate your feedback, which has helped us strengthen the quality and relevance of the paper.

**2.** **Lines 104–114: The review on the role of counting in numerical mapping is limited, making the rationale for conducting Experiment 2 relatively weak. The necessity of this experiment is not strongly justified. It is advised to expand the discussion on counting-related research to provide a more solid foundation for this experimental design.**
**Response：**Thank you for your insightful comment. In response, we have reorganized the *Introduction* section with clear subheadings. Specifically, we have added a dedicated section on the "Effect of Counting on Numerical Mapping," in which we have systematically reviewed relevant studies, summarized previous findings in greater detail, and elaborated on the necessity and rationale for conducting Experiment 2. These revisions strengthen the theoretical foundation of our experimental design (see Pages 8-9, Lines 148–183).

**3. There are methodological concerns regarding the experimental setup. Line 201: Participants completed tasks in the order presented, which raises the possibility that increased familiarity with the tasks could lead to improved performance in later trials or restrict certain pathways. We suggest that the task order be randomized to obtain more reliable and generalizable conclusions. Additionally, the participants in the two experiments were not the same, introducing the possibility that initial differences between the groups may have influenced the results. Specifically, after introducing counting as a variable, the source of the observed differences cannot be conclusively determined, as this was not addressed in the methodology. Controlling for initial variables would enhance the validity of the findings.**
**Response：**Thank you very much for your insightful comments regarding the experimental design and potential practice effects. As the study was conducted using paper-based materials bound into booklets (as in Jiménez Lira et al., 2017), the trial order had to be fixed to allow the experimenter to accurately record their responses. We acknowledge that this fixed sequence might introduce order or practice effects. To minimize such influence, we adopted a Latin square design for the presentation of the target numbers (small numbers: 1, 2, 3, 2, 3, 1; large numbers: 4, 5, 6, 5, 4, 6), and the positions of the six alternative choices (arrays and digits) in the four mapping tasks were randomized and counterbalanced across trials.

In addition, different groups of participants were recruited for the two experiments. This between-subjects design was deliberately employed to avoid potential carryover or learning effects that might occur if the same children completed both experimental conditions. Nevertheless, we recognize that using different participants may introduce variability due to individual differences, and this is indeed a limitation of our study.

We have now explicitly acknowledged these limitations and their implications in the revised manuscript (see Page 32, Lines 700–705).

**Revised Text in Manuscript:** “This study has several limitations. First, the participants were recruited from three towns across two provinces, which may limit the generalizability of the findings. The study also employed a cross-sectional design with a fixed task order, potentially introducing order effects and individual variability. Future research should consider broader sampling, longitudinal tracking, and fully randomized within-subject designs to enhance generalizability and experimental control.”

**4. Line 270: The comparison drawn here appears somewhat arbitrary. Since the data points are not from the same temporal context, they may be influenced by differences in educational background and broader societal developments over time. It is recommended that this comparison be removed or revised to present a more rigorous interpretation.**

**Response：**Thank you very much for your thoughtful comment. We fully agree that direct comparisons between different studies should be made with caution, especially when participants may differ in educational or sociocultural background. In response to a similar suggestion from another reviewer, we have revised the relevant paragraph to avoid making strong claims based on cross-study comparisons (see Page 18, Lines 372–376). The revised text now carefully describes the performance differences observed between our study and that of Benoit et al. (2013), while also explicitly acknowledging that such differences may reflect variability in children’s numerical development across samples or differences in educational or cultural context, rather than conclusive developmental patterns. We appreciate your valuable feedback, which has helped us improve the rigor of our interpretation.

**Revised Text in Manuscript:** “These results differ from those reported by Benoit et al. (2013), in which 3-year-old children’s performance on most mapping paths was below chance. While both studies used similar materials and procedures, the performance differences may reflect variability in children’s numerical development across samples or differences in educational or cultural context.”
